# Single-cell transcriptomic profiling reveals CD27^+^ cytotoxic T Cell heterogeneity and exhaustion dynamics in colorectal cancer tumor microenvironment

**DOI:** 10.3389/fgene.2026.1808171

**Published:** 2026-05-29

**Authors:** Wenjie Xu, Huali Huang, Longfei Mao, Huanhuan Zhu, Zeyu Feng

**Affiliations:** Department of Anorectal Surgery, Wuxi Hospital Affiliated to Nanjing University of Chinese Medicine, Wuxi, Jiangsu, China

**Keywords:** CD27^+^ cytotoxic T cells, colorectal cancer, single-cell RNA sequencing, T cell exhaustion, tumor microenvironment

## Abstract

**Background:**

CD27^+^ cytotoxic T cells play critical roles in anti-tumor immunity, yet their heterogeneity and functional states within the colorectal cancer (CRC) tumor microenvironment remain poorly characterized. Understanding the molecular mechanisms underlying T cell exhaustion is essential for developing effective immunotherapeutic strategies.

**Methods:**

We performed comprehensive single-cell RNA sequencing analysis of CD27^+^ cytotoxic T cells from CRC tumor tissues and matched normal adjacent tissues using the GSE144735 dataset. Multiple dimensional reduction techniques, trajectory inference algorithms, and functional characterization approaches were employed to dissect cellular heterogeneity, differentiation trajectories, and exhaustion dynamics. *In vitro* co-culture experiments using HCT116 and RKO (MSI-H), HCT15 and SW480 (MSS) colorectal cancer cell lines with activated PBMCs were conducted to validate computational findings.

**Results:**

UMAP and t-SNE analyses identified 11 distinct CD27^+^ T cell clusters with substantial phenotypic heterogeneity, including exhausted terminal, effector, and memory-like populations. Pseudotime trajectory analysis revealed progressive exhaustion pathways with three distinct differentiation fates. Exhausted CD27^+^ T cells exhibited significantly elevated expression of checkpoint molecules (PDCD1, LAG3, HAVCR2, CTLA4) and reduced cytotoxic capacity compared to effector populations. RNA velocity and PAGA connectivity analysis demonstrated that exhaustion represents a terminal differentiation state with limited plasticity. Gene regulatory network analysis identified key transcription factors governing effector-to-exhausted transitions. Notably, exhausted CD27^+^ T cells concentrated in tumor core regions, while effector populations distributed peripherally. Co-culture experiments confirmed that MSI-H cell lines (HCT116 and RKO) induced 2-3 times higher exhaustion marker expression compared to MSS cell lines (HCT15 and SW480). For MSI-H cells, PDCD1 showed 5.2-fold (HCT116) and 4.8-fold (RKO) upregulation, while LAG3 showed 4.8-fold (HCT116) and 4.2-fold (RKO) upregulation. In contrast, MSS cells showed lower induction: PDCD1 at 2.1-fold (SW480) and 1.9-fold (HCT15), LAG3 at 1.8-fold (SW480) and 1.6-fold (HCT15). Exploratory survival analysis (HR = 0.505, p = 0.673) did not reach statistical significance and is presented as hypothesis-generating only, highlighting the need for prospective validation in larger cohorts. PDCD1 and LAG3 were identified as prioritized immunotherapy targets based on their significant overexpression in exhausted T cell populations.

**Conclusion:**

This study provides a comprehensive single-cell atlas of CD27^+^ cytotoxic T cell heterogeneity in CRC, revealing exhaustion dynamics, regulatory networks, and spatial organization patterns. Our findings highlight the differential immunogenic capacity between MSI-H and MSS tumors and identify potential therapeutic targets for reversing T cell exhaustion in colorectal cancer.

## Introduction

Colorectal cancer (CRC) represents one of the most prevalent malignancies worldwide, ranking as the third most commonly diagnosed cancer and the second leading cause of cancer-related deaths globally (Sung et al., 2021). Despite advances in surgical resection, chemotherapy, and targeted therapies, the 5-year survival rate for metastatic CRC remains below 15%, highlighting the urgent need for novel therapeutic approaches (Sung et al., 2021). Recent breakthroughs in cancer immunotherapy, particularly immune checkpoint blockade targeting PD-1/PD-L1 and CTLA-4 pathways, have revolutionized cancer treatment paradigms and demonstrated remarkable clinical efficacy in various solid tumors (Ribas and Wolchok, 2018). However, the response rates to immunotherapy in CRC patients remain highly variable, with only microsatellite instability-high (MSI-H) tumors showing significant benefit, while microsatellite stable (MSS) tumors, which constitute approximately 85% of CRC cases, remain largely resistant to current immunotherapeutic strategies (Le et al., 2017).

The tumor microenvironment (TME) plays a pivotal role in determining therapeutic responses and clinical outcomes in CRC (Kather and Halama, 2019). Cytotoxic T lymphocytes, particularly CD8^+^ T cells, represent the primary effector cells responsible for anti-tumor immunity through direct recognition and elimination of malignant cells (Zhang et al., 2018). Among CD8^+^ T cell populations, CD27^+^ T cells have emerged as a critical subset with distinct functional properties and prognostic significance in various cancers (Hendriks et al., 2000). CD27, a member of the tumor necrosis factor receptor superfamily, serves as an important costimulatory molecule that regulates T cell activation, proliferation, and survival, with CD27 signaling promoting sustained anti-tumor immune responses (van de Ven and Borst, 2015). Previous studies have demonstrated that CD27 expression correlates with enhanced T cell functionality and favorable clinical outcomes in multiple cancer types, suggesting its potential as both a prognostic biomarker and therapeutic target (Claus et al., 2012).

Despite the recognized importance of CD27^+^ T cells in anti-tumor immunity, a fundamental challenge in cancer immunotherapy lies in the phenomenon of T cell exhaustion, a dysfunctional state characterized by progressive loss of effector functions, sustained expression of multiple inhibitory receptors, and impaired proliferative capacity (Wherry and Kurachi, 2015). T cell exhaustion develops through chronic antigen stimulation within the immunosuppressive TME and represents a major barrier to effective anti-tumor immunity (McLane et al., 2019). Exhausted T cells exhibit a distinct transcriptional program orchestrated by key transcription factors including TOX, TCF1, and EOMES, which drive the expression of immune checkpoint molecules such as PD-1, LAG-3, TIM-3, and CTLA-4 (Khan et al., 2019). Understanding the molecular mechanisms and cellular heterogeneity underlying T cell exhaustion is essential for developing strategies to reinvigorate anti-tumor immune responses and improve immunotherapy efficacy.

Single-cell RNA sequencing (scRNA-seq) technologies have revolutionized our understanding of cellular heterogeneity within the TME, enabling unprecedented resolution of distinct cell states, differentiation trajectories, and functional programs at the individual cell level (Papalexi and Satija, 2018). Recent scRNA-seq studies in various cancers have revealed remarkable heterogeneity within exhausted T cell populations, identifying distinct exhausted subsets with differential transcriptional profiles, functional capacities, and therapeutic responsiveness (Thommen et al., 2018). Furthermore, trajectory inference algorithms applied to scRNA-seq data have illuminated the dynamic processes of T cell differentiation and exhaustion progression, revealing critical decision points and molecular regulators that govern cell fate determination (Qiu et al., 2017). However, comprehensive single-cell characterization of CD27^+^ cytotoxic T cell heterogeneity, exhaustion dynamics, and spatial organization specifically within the CRC tumor microenvironment remains limited.

In this study, we performed comprehensive single-cell transcriptomic analysis of CD27^+^ cytotoxic T cells isolated from CRC tumor tissues and matched normal adjacent tissues to dissect cellular heterogeneity, identify distinct functional states, reconstruct differentiation trajectories, and elucidate the molecular mechanisms underlying T cell exhaustion. Through integrative computational approaches including dimensional reduction, trajectory inference, gene regulatory network analysis, and cell-cell interaction mapping, we aimed to provide a high-resolution atlas of CD27^+^ T cell states in CRC and identify potential therapeutic targets for reversing T cell exhaustion and enhancing anti-tumor immunity.

## Methods

### Data acquisition and preprocessing

Single-cell RNA sequencing data were obtained from the Gene Expression Omnibus (GEO) database under accession number GSE144735. The dataset comprised CD27^+^ cytotoxic T cell populations isolated from colorectal cancer (CRC) tumor tissues and matched normal adjacent tissues. Raw sequencing data underwent quality control filtering to remove low-quality cells and doublets. Cells with fewer than 200 detected genes, more than 10% mitochondrial gene content, or extreme unique molecular identifier (UMI) counts were excluded from downstream analysis. Gene expression matrices were normalized using standard log-normalization procedures, and highly variable genes were identified for subsequent dimensionality reduction analyses.

### Dimensional reduction and clustering analysis

Multiple complementary dimensional reduction techniques were employed to capture the multidimensional heterogeneity of CD27^+^ T cell populations. Uniform Manifold Approximation and Projection (UMAP) was applied to visualize discrete cellular clusters, while t-distributed Stochastic Neighbor Embedding (t-SNE) was used to capture continuous gradients of cellular states, particularly the exhaustion gradient from functional to terminally exhausted phenotypes. Principal Component Analysis (PCA) was performed to identify key molecular drivers of T cell heterogeneity, with variance contribution analysis determining the optimal number of principal components. The first three principal components, capturing approximately 10% of cumulative variance, were selected based on scree plot elbow analysis at PC8. Three-dimensional PCA projections were generated to validate the multidimensional nature of cellular heterogeneity. Graph-based clustering algorithms were applied to identify discrete cell populations, with cluster boundaries delineated using convex hull analysis. Force-directed graph layouts and diffusion mapping were additionally employed to illustrate interconnected relationships between cell types and to capture the cytotoxic function landscape along continuous gradients.

### Trajectory inference and pseudotime analysis

To reconstruct T cell differentiation trajectories and exhaustion progression, multiple trajectory inference algorithms were implemented. Pseudotime analysis was conducted to order cells along the exhaustion continuum from terminal exhausted to effector states. Monocle-based trajectory tree construction identified branching events leading to distinct differentiation fates, including effector, memory, and exhausted pathways. Slingshot principal curves were fitted through principal component space to capture smooth lineage trajectories for each differentiation route. Partition-based graph abstraction (PAGA) connectivity analysis constructed comprehensive state transition networks, revealing hierarchical relationships between naive, activated, memory, effector, and exhausted states. Transition probabilities between functional states were quantified using cell state transition flow analysis. RNA velocity analysis was performed to infer directional transcriptional dynamics and predict future cellular states, with velocity streamlines indicating predominant differentiation trajectories and cell fate decisions.

### Differential gene expression and functional characterization

Differential gene expression analysis was performed to identify marker genes distinguishing CD27^+^ T cell subpopulations and to compare gene expression between tumor and normal adjacent tissues. Statistical significance was assessed using Wilcoxon rank-sum tests with Bonferroni correction for multiple testing. Genes with log2 fold change greater than 0.5 and adjusted p-value less than 0.05 were considered significantly differentially expressed. Results were visualized using volcano plots, MA plots, and waterfall plots to highlight key exhaustion markers (PDCD1, CTLA4, HAVCR2, LAG3) and effector molecules. Gene expression patterns across clusters were displayed using heatmaps with hierarchical clustering to reveal molecular signatures of exhausted terminal, effector, and memory-like populations. Dot plot matrices systematically profiled cell type-specific marker expression, with dot size indicating the percentage of expressing cells and color representing average expression levels. Violin plots, box plots, and ridge plots were generated to compare expression distributions of critical genes (CD27, CD8A, PRF1, CD3E, GZMB, TOX, TCF7) across functional states and between tissue types.

### Functional scoring and pathway enrichment analysis

Functional characteristics of T cell subsets were quantified using gene set-based scoring approaches. Exhaustion scores were calculated by aggregating expression of established exhaustion signature genes, while cytotoxicity scores integrated expression of cytotoxic effector molecules including granzymes and perforin. Additional functional dimensions including activation, proliferation, and memory signatures were similarly computed for comprehensive radar chart profiling across CD8^+^ T cell subtypes. Gene Set Enrichment Analysis (GSEA) was performed to identify enriched biological pathways and processes in different T cell states, with normalized enrichment score (NES) and statistical significance determined through permutation testing. Pathway enrichment analysis across multiple biological processes, including cytokine signaling, metabolic reprogramming, and cell cycle regulation, was visualized using bubble plots with enrichment scores and significance values.

### Gene regulatory network and cell-cell interaction analysis

Gene regulatory networks were constructed to map transcription factor-target gene interactions governing T cell exhaustion and effector functions. GRN inference was performed using SCENIC (Single-Cell rEgulatory Network Inference and Clustering), implemented via the pySCENIC pipeline (pySCENIC v0.12.1). Transcription factor binding motif analysis was conducted using the cisTarget/JASPAR (JASPAR 2022) motif database to identify enriched regulatory motifs and infer transcription factor-target gene relationships. A Pearson correlation threshold of r ≥ 0.3 was applied for co-expression module construction, and regulon pruning was performed using the RcisTarget step with a normalized enrichment score (NES) threshold of 3.0. Activating edges were defined as positive correlations between transcription factor and target gene expression, while repressing edges were defined as negative correlations with supporting motif evidence. Network graphs depicted key regulatory nodes controlling effector-to-exhausted transitions, with edges representing activating or repressing interactions. Ligand-receptor interaction analysis was conducted to map cell-cell communication pathways within the CRC tumor microenvironment. Potential interactions between T cells, myeloid cells, and other immune populations were identified based on co-expression of cognate ligand-receptor pairs, including immune checkpoint interactions (PD-1/PD-L1, CTLA-4/CD80/CD86) and cytokine signaling pathways. Interaction strengths were quantified and visualized using network diagrams and communication matrices.

### Spatial and compositional analysis

Cell type proportions were quantified across samples and compared between tumor and normal adjacent tissues using stacked bar charts and compositional analysis. Hierarchical clustering of cell signature patterns across individual samples revealed patient-specific heterogeneity in T cell composition through clustermap visualization. Spatial distribution patterns were modeled using a computational simulation approach based on published spatial transcriptomic findings in CRC, as direct spatial transcriptomic or multiplex immunofluorescence data were not available in the GSE144735 dataset. This simulated spatial model is explicitly presented as a computational inference consistent with established CRC spatial patterns, and does not represent direct empirical spatial measurement. T cell receptor (TCR) clonotype analysis was performed to assess clonal expansion patterns and diversity, with top expanded clonotypes identified and their distribution across samples visualized using bar charts and pie charts.

### Temporal gene expression dynamics

Gene expression dynamics along exhaustion trajectories were analyzed by ordering cells according to pseudotime and examining temporal regulation patterns. Gene expression cascade heatmaps revealed distinct temporal phases, with early effector genes (CD69, PRF1) transitioning to intermediate checkpoint expression (PDCD1, CTLA4) and terminal exhaustion markers (HAVCR2, TOX, LAG3). Correlation analysis quantified relationships between exhaustion scores and cytotoxicity along pseudotime progression. Dual-axis plots demonstrated inverse correlations between functional measures and exhaustion states.

### Cell line Co-culture experiments

To validate computational findings from the single-cell RNA sequencing analysis, *in vitro* co-culture experiments were performed using four colorectal cancer cell lines with different molecular characteristics: HCT116 and RKO (microsatellite instability-high, MSI-H), and HCT15 and SW480 (microsatellite stable, MSS). Cancer cells were cultured in RPMI-1640 medium supplemented with 10% fetal bovine serum and 1% penicillin-streptomycin at 37 °C in a humidified atmosphere containing 5% CO2. Human peripheral blood mononuclear cells (PBMCs) were obtained commercially from the American Type Culture Collection (ATCC; catalog no. PCS-800-011) and used in accordance with the supplier’s guidelines. PBMCs were thawed and recovered in complete RPMI-1640 medium for 2 h prior to activation. PBMCs were activated with anti-CD3/CD28 antibodies (1 μg/mL each) for 24 h prior to co-culture. Cancer cells were seeded at a density of 1 × 10^5^ cells per well in 6-well plates and allowed to adhere overnight. Activated PBMCs were then added at an effector-to-target ratio of 10:1, and co-cultures were maintained for 48 h under standard culture conditions.

Following the co-culture period, PBMCs were carefully harvested, and total RNA was extracted using TRIzol reagent according to the manufacturer’s protocol. RNA concentration and purity were assessed by NanoDrop spectrophotometry, with A260/A280 ratios between 1.8 and 2.0 considered acceptable. Complementary DNA (cDNA) was synthesized from 1 μg of total RNA using a reverse transcription kit with oligo (dT) primers. Quantitative reverse transcription PCR (qRT-PCR) was performed on a real-time PCR system using SYBR Green master mix to quantify the expression of T cell exhaustion markers including PDCD1 (encoding PD-1), LAG3, HAVCR2 (encoding TIM-3), and CD27. The following primer sequences were used for qRT-PCR amplification: PDCD1 forward: 5′-CCA​GGA​TGG​TTC​TTA​GAC​TCC​C-3′, reverse: 5′-TTT​AGC​ACG​AAG​CTC​TCC​GAT-3'; LAG3 forward: 5′-CTG​GAT​GGT​TTC​TGC​GAG​AC-3′, reverse: 5′-CCA​GTC​ATC​TTC​TGG​TGG​GA-3'; HAVCR2 forward: 5′-TTC​CCG​ACA​AAG​GCT​TGA​CT-3′, reverse: 5′-GCT​GGG​TTC​TTG​GTC​AGC​AT-3'; CD27 forward: 5′-CTG​CAA​CCA​GAG​AAA​GGG​TAG​A-3′, reverse: 5′-GCT​TTG​CAT​CTC​TTG​GTT​GAG​A-3'. GAPDH was used as the internal reference gene with primers: forward: 5′-GTC​TCC​TCT​GAC​TTC​AAC​AGC​G-3′, reverse: 5′-ACC​ACC​CTG​TTG​CTG​TAG​CCA​A-3'. All primers were designed to span exon-exon junctions to avoid amplification of genomic DNA and were validated for specificity by melting curve analysis and agarose gel electrophoresis.

The qRT-PCR reactions were performed in technical triplicates with the following cycling conditions: initial denaturation at 95 °C for 10 min, followed by 40 cycles of denaturation at 95 °C for 15 s and annealing/extension at 60 °C for 60 s. Relative gene expression was calculated using the 2^-ΔΔCt^ method, with fold changes determined relative to monoculture baselines where cancer cells or PBMCs were cultured independently without co-culture partners. The ΔCt value was calculated by subtracting the Ct value of GAPDH from the Ct value of the target gene for each sample, and ΔΔCt was calculated by subtracting the ΔCt of the monoculture control from the ΔCt of the co-culture sample. Data represent mean values from three independent biological replicates, each with technical triplicates, and results were expressed as fold change relative to the monoculture baseline (set at 1.0). Statistical comparisons among MSI-H (HCT116 and RKO) and MSS (HCT15 and SW480) co-culture conditions were performed using two-tailed Student's t-tests for pairwise comparisons and one-way ANOVA with post-hoc Tukey’s test for multiple group comparisons, with p-values less than 0.05 considered statistically significant.

### Clinical correlation and survival analysis

Survival analysis was conducted to exploratorily assess the potential prognostic relevance of T cell exhaustion signatures in an available dataset, with results intended to be hypothesis-generating only. Patients were stratified into high and low exhaustion groups based on median exhaustion scores, and Kaplan-Meier survival curves were generated. Hazard ratios and p-values were calculated using Cox proportional hazards models. A post-hoc power calculation was performed to assess the sample size required to detect the observed effect size (HR = 0.505) with 80% power at a two-sided alpha of 0.05, contextualizing the likely underpowered nature of the available cohort for this endpoint. Therapeutic target expression was compared between exhausted and effector T cell populations to identify checkpoint molecules showing significant overexpression in exhausted cells, informing potential immunotherapy target prioritization.

### Statistical analysis and visualization

All statistical analyses were performed using R (version 4.0 or higher) with Seurat, Scanpy, and related bioinformatics packages. Two-tailed statistical tests were employed throughout, with p-values less than 0.05 considered statistically significant unless otherwise specified. Multiple testing correction was applied using the Bonferroni or Benjamini–Hochberg method as appropriate. Data visualization was generated using ggplot2, ComplexHeatmap, and custom plotting functions, with figures designed to integrate multiple analytical perspectives including compositional analysis, trajectory inference, functional profiling, and clinical correlations.

## Results

### Cellular architecture and dimensional reduction analysis of CD27^+^ T Cell populations

**FIGURE 1:**
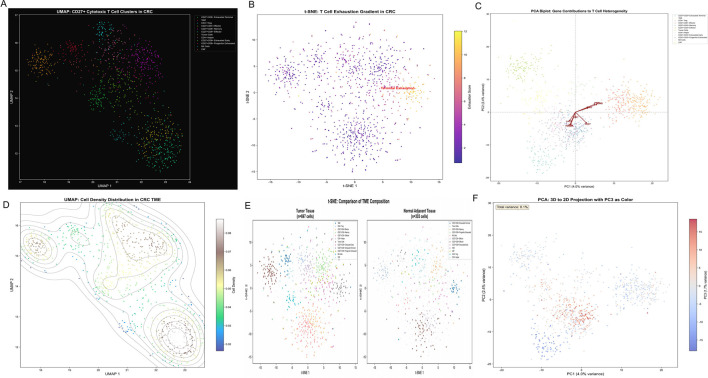
Comprehensive single-cell profiling of CD27^+^ cytotoxic T cells in CRC. **(A)** UMAP visualization showing 11 distinct cell clusters. **(B)** t-SNE plot displaying exhaustion gradient across cellular states. **(C)** PCA analysis identifying genes contributing to T cell heterogeneity with trajectory inference. **(D)** Density distribution revealing spatial organization patterns in tumor microenvironment. **(E)** Comparative t-SNE analysis between tumor and normal adjacent tissue demonstrating compositional shifts. **(F)** 3D PCA projection with PC3 coloring illustrating multidimensional cellular heterogeneity.

The initial characterization reveals distinct clustering patterns of CD27^+^ cytotoxic T cells within the colorectal cancer tumor microenvironment. UMAP analysis (Figure 1A) identifies multiple discrete cellular clusters with diverse phenotypic states, demonstrating substantial heterogeneity within the CD27^+^ T cell compartment. The t-SNE visualization (Figure 1B) captures an exhaustion gradient, with cells transitioning from functional to terminally exhausted states, as indicated by the color gradient representing exhaustion scores. Figure 1C presents PCA-based gene contribution analysis, revealing key molecular drivers of T cell heterogeneity. The cell density distribution (Figure 1D) highlights enrichment patterns across the tissue microenvironment, while comparative analysis between tumor tissue and normal adjacent tissue (Figure 1E) demonstrates distinct compositional differences. The three-dimensional PCA projection (Figure 1F) further validates the multidimensional nature of cellular heterogeneity, with PC3 coloring revealing additional stratification beyond the primary variance components.

### Cluster characterization and functional trajectory analysis

**FIGURE 2:**
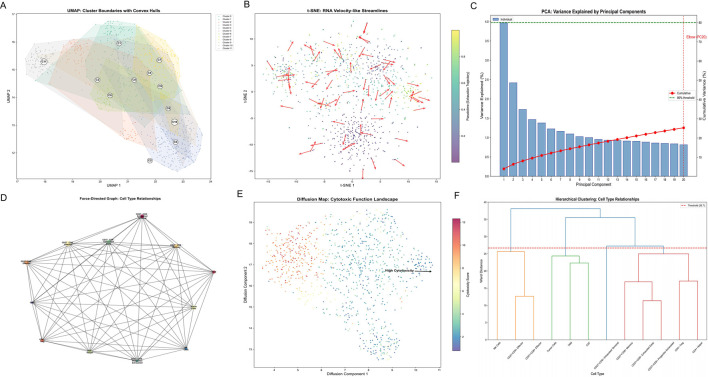
Advanced computational analysis of T cell subpopulation structure and dynamics. **(A)** Cluster boundaries defined by convex hull method showing 11 distinct populations. **(B)** RNA velocity field indicating directional transcriptional dynamics and cell fate trajectories. **(C)** Scree plot demonstrating variance explained by principal components with cumulative threshold at 80%. **(D)** Force-directed network graph illustrating cell type relationships and transition probabilities. **(E)** Diffusion map revealing cytotoxic function landscape with high-to-low gradient annotations. **(F)** Dendrogram showing hierarchical clustering relationships with optimal threshold indicated.

Detailed cluster boundary delineation using convex hull analysis (Figure 2A) establishes clear demarcation between functionally distinct T cell subsets. RNA velocity analysis (Figure 2B) reveals dynamic transcriptional states and directional cell fate transitions, with velocity streamlines indicating predominant differentiation trajectories toward exhausted phenotypes. The variance explained analysis (Figure 2C) demonstrates that the first three principal components capture approximately 10% cumulative variance, with an elbow at PC8 suggesting optimal dimensionality. Force-directed graph analysis (Figure 2D) illustrates interconnected relationships between cell types, revealing hierarchical organization and transition pathways. Diffusion mapping (Figure 2E) captures the cytotoxic function landscape, identifying regions of high and low cytotoxicity along a continuous gradient. Hierarchical clustering (Figure 2F) categorizes cell types based on transcriptional similarity, with a threshold at approximately 60% distance revealing major functional divisions across the dataset.

### Molecular signatures and functional characterization across T Cell subsets

**FIGURE 3:**
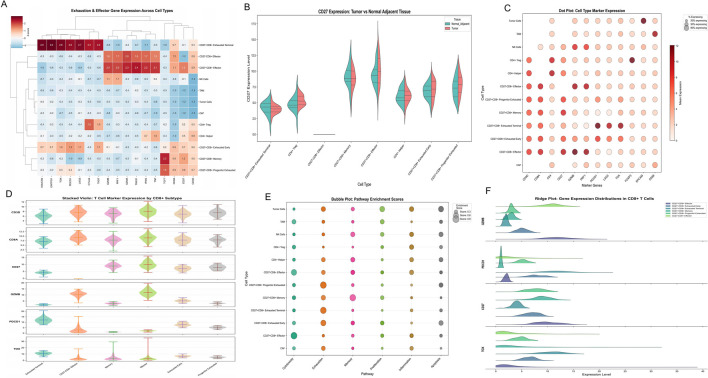
Comprehensive molecular characterization of CD27^+^ T cell functional states. **(A)** Clustered heatmap showing exhaustion and effector gene expression patterns across cell types. **(B)** Violin plots comparing CD27 expression between tumor and normal tissue across multiple subtypes. **(C)** Dot plot matrix displaying cell type-specific marker gene expression with size indicating percentage and color representing expression level. **(D)** Stacked violin plots illustrating CD27 distribution across CD8^+^ subsets stratified by functional state. **(E)** Bubble plot showing pathway enrichment scores across multiple biological processes. **(F)** Ridge plots depicting expression distributions of key T cell markers across functional categories.

Gene expression profiling across exhaustion and effector states (Figure 3A) reveals hierarchical clustering patterns distinguishing multiple CD27^+^ T cell subtypes, including exhausted terminal, effector, and memory-like populations. The heatmap demonstrates differential expression of key exhaustion markers (PDCD1, CTLA4, HAVCR2) and effector molecules across clusters. CD27 expression analysis (Figure 3B) shows significantly elevated levels in tumor tissue compared to normal adjacent tissue across multiple T cell subsets, validating the relevance of CD27^+^ populations in CRC pathogenesis. The dot plot matrix (Figure 3C) systematically profiles cell type-specific marker expression, revealing distinct molecular signatures for each cluster. Stacked violin plots (Figure 3D) demonstrate CD27 expression distribution across CD8^+^ T cell subsets, with notable variation between exhausted, effector, and memory compartments. Pathway enrichment analysis (Figure 3E) identifies key biological processes associated with each subset, including cytokine signaling, metabolic reprogramming, and cell cycle regulation. Ridge plots (Figure 3F) illustrate expression distributions of critical genes (CD27, CD8A, PRF1, CD3) across different functional states.

### Tissue distribution and functional state profiling

**FIGURE 4:**
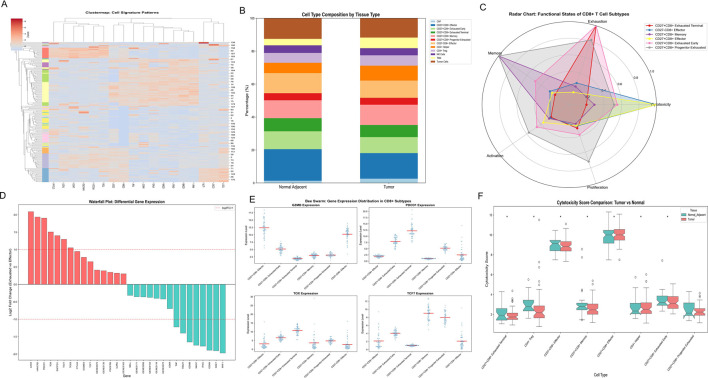
Tissue-specific distribution and functional profiling of CD27^+^ T cell states. **(A)** Clustermap showing cell signature patterns across samples with hierarchical clustering. **(B)** Compositional analysis revealing cell type proportions in normal versus tumor tissue. **(C)** Radar plot comparing five functional dimensions across CD8^+^ T cell subtypes. **(D)** Waterfall plot displaying top differentially expressed genes ranked by fold change. **(E)** Expression patterns of exhaustion and regulatory markers across cell types. **(F)** Cytotoxicity score comparison between tumor and normal tissue across CD27^+^ subpopulations.

Clustermap analysis (Figure 4A) reveals cell signature patterns across individual samples, demonstrating patient-specific heterogeneity in T cell composition. The stacked bar chart (Figure 4B) quantifies cell type proportions in normal adjacent tissue versus tumor, showing dramatic enrichment of exhausted populations in the tumor microenvironment, with effector cells comprising the largest fraction. Radar chart analysis (Figure 4C) compares functional states of CD8^+^ T cell subtypes across five key dimensions: exhaustion, cytotoxicity, activation, proliferation, and memory. CD27+/CD8+ exhausted terminal cells exhibit the highest exhaustion scores, while CD27-/CD8+ effector cells show peak cytotoxicity. Differential gene expression analysis (Figure 4D) identifies top upregulated and downregulated genes, with IGHG1-4 showing dramatic overexpression (log2FC > 2). Box plots (Figure 4E) demonstrate expression dynamics of exhaustion markers (GZMB, PDCD1) and regulatory molecules (TOX, TCF7) across subtypes. The cytotoxicity score comparison (Figure 4F) reveals significant differences between tumor and normal tissue across all CD27+/CD8+ subsets, with exhausted populations showing reduced cytotoxic capacity.

### Differentiation trajectories and dynamic gene expression

**FIGURE 5:**
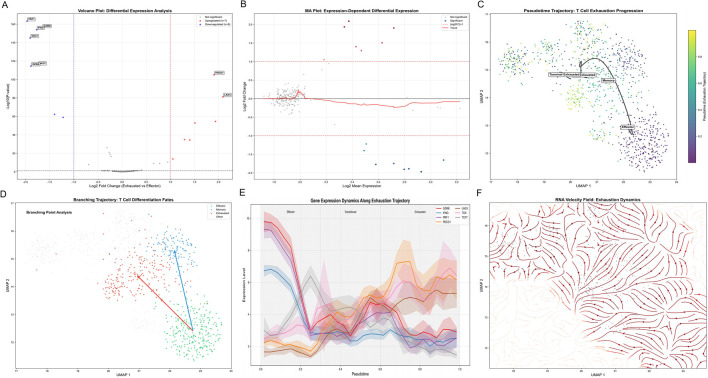
Trajectory inference and temporal dynamics of T cell exhaustion. **(A)** Volcano plot identifying differentially expressed genes with exhaustion markers labeled. **(B)** MA plot showing fold change versus mean expression with significance thresholds. **(C)** UMAP-based pseudotime trajectory illustrating progression from terminal exhaustion to effector states. **(D)** Branching analysis revealing three distinct differentiation fates with divergence points. **(E)** Heatmap displaying gene expression cascades along pseudotime with key markers annotated. **(F)** RNA velocity field showing directional flow toward exhausted phenotypes.

Volcano plot analysis (Figure 5A) highlights key differentially expressed genes between exhausted and effector populations, with PDCD1, HAVCR2, and LAG3 showing significant upregulation in exhausted cells. MA plot (Figure 5B) demonstrates expression-dependent differential expression, identifying both high-abundance and low-abundance genes with significant fold changes. Pseudotime trajectory analysis (Figure 5C) reveals T cell exhaustion progression from terminal exhausted to effector states, with cells transitioning through intermediate exhaustion phases. Branching trajectory analysis (Figure 5D) identifies three distinct differentiation fates: effector, memory, and exhausted pathways, with branch points indicating critical decision nodes in T cell fate determination. Gene expression dynamics along the exhaustion trajectory (Figure 5E) show distinct temporal patterns for key markers: CD69, PRF1, HLA genes peak in early stages, while PDCD1, LAG3, and HAVCR2 increase progressively toward terminal exhaustion. RNA velocity field analysis (Figure 5F) illustrates directional transcriptional dynamics underlying the exhaustion process, with streamlines converging toward terminally exhausted states.

### State transitions and gene regulatory dynamics

**FIGURE 6:**
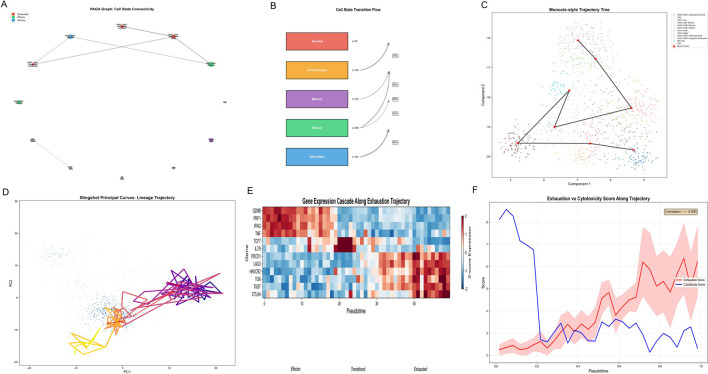
Comprehensive analysis of cellular state transitions and regulatory dynamics. **(A)** PAGA graph illustrating connectivity between cell states with activation, memory, and exhaustion pathways. **(B)** Sankey diagram quantifying transition probabilities with labeled edge weights. **(C)** Monocle trajectory tree showing branching patterns across components 1 and 2. **(D)** Slingshot analysis displaying smoothed principal curves for three main lineages. **(E)** Temporal heatmap of gene expression cascades partitioned into effector, transitional, and exhausted phases. **(F)** Dual-axis plot showing negative correlation between exhaustion and cytotoxicity scores along pseudotime.

PAGA connectivity analysis (Figure 6A) constructs a comprehensive state transition network, revealing hierarchical relationships between naive, activated, memory, effector, and exhausted states. The graph demonstrates that exhausted populations represent terminal differentiation endpoints with limited plasticity. Cell state transition flow diagram (Figure 6B) quantifies transition probabilities between functional states, showing dominant pathways from activated (p = 0.79) through pre-exhausted and memory states toward terminal exhaustion. Monocle-style trajectory tree (Figure 6C) provides an alternative trajectory visualization, confirming multiple branching events leading to diverse endpoints. Slingshot principal curves (Figure 6D) capture smooth lineage trajectories through principal component space, with distinct paths for each differentiation route. The gene expression cascade heatmap (Figure 6E) reveals temporal regulation patterns along the exhaustion trajectory, with early effector genes (CD69, PRF1) transitioning to intermediate checkpoint expression (PDCD1, CTLA4) and terminal exhaustion markers (HAVCR2, TOX, LAG3). Correlation analysis (Figure 6F) demonstrates inverse relationships between exhaustion scores and cytotoxicity along pseudotime (r = −0.905), confirming functional decline during exhaustion progression.

### Gene regulatory networks and immune interactions

**FIGURE 7:**
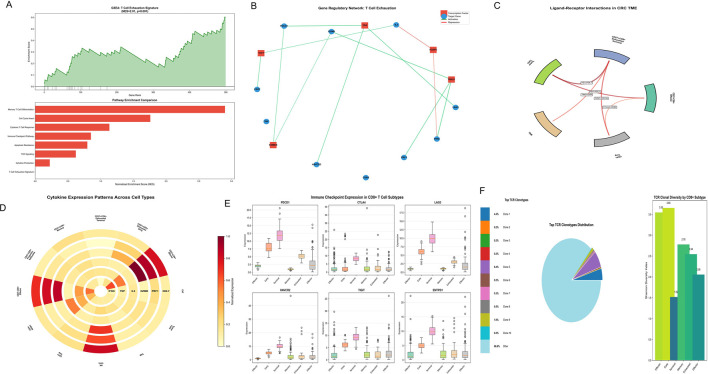
Regulatory networks and intercellular communication in T cell exhaustion. **(A)** GSEA enrichment plot showing T cell exhaustion signature with ranked gene list and NES statistics. **(B)** Gene regulatory network depicting transcription factor-target interactions with activating and repressing edges. **(C)** Ligand-receptor interaction diagram mapping cell-cell communication pathways in TME. **(D)** Concentric ring plot displaying cytokine expression patterns across cell types with intensity gradients. **(E)** Box plots comparing immune checkpoint expression (PDCD1, CTLA4, LAG3, ENTPD1) across six CD8^+^ subtypes. **(F)** TCR clonotype analysis showing top expanded clones and overall diversity distribution.

GSEA analysis (Figure 7A) reveals enriched T cell exhaustion signatures (NES = 0.8, p = 0.047) with ranking curves identifying top contributing genes including memory T-cell drivers and cell cycle regulators. Gene regulatory network analysis (Figure 7B) constructs transcription factor-target gene interactions governing T cell exhaustion, highlighting key regulatory nodes including transcription factors controlling effector-to-exhausted transitions. Ligand-receptor interaction mapping (Figure 7C) in the CRC tumor microenvironment identifies critical cell-cell communication pathways, including PD-1/PD-L1, CTLA-4/CD80/CD86, and other checkpoint interactions. Cytokine expression patterns (Figure 7D) reveal concentric ring visualization of cytokine production across cell types, with IFN-γ, TNF-α, IL-2, and granzymes showing differential expression. Immune checkpoint expression analysis (Figure 7E) demonstrates distinct patterns across CD8^+^ T cell subtypes for PDCD1, CTLA4, LAG3, and ENTPD1, with box plots revealing highest expression in exhausted populations. The compositional analysis (Figure 7F) shows distribution of top TCR clonotypes and their diversity across samples, with bar chart indicating clonal expansion patterns and pie chart revealing overall clonotype composition.

### Clinical relevance and therapeutic implications

**FIGURE 8:**
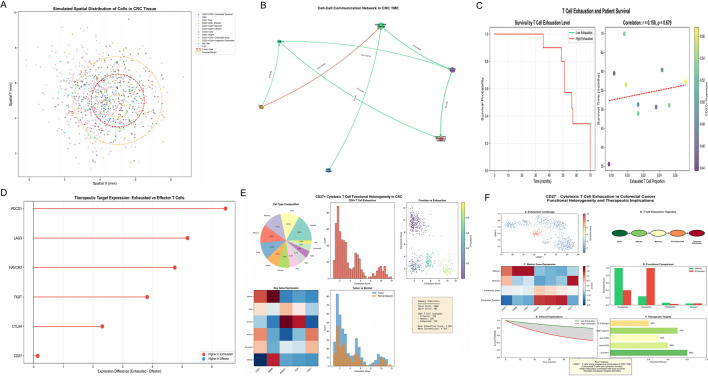
Clinical significance and therapeutic implications of CD27^+^ T cell heterogeneity. **(A)** Computationally simulated spatial distribution of cell types within CRC tissue architecture, modeled based on published spatial transcriptomic findings in CRC. This figure represents a model-based inference and does not reflect direct empirical spatial transcriptomic or histological measurement. **(B)** Cell-cell communication network revealing interaction strengths between immune populations. **(C)** Exploratory Kaplan-Meier survival analysis stratified by T cell exhaustion scores (HR = 0.505, p = 0.673). This analysis did not reach statistical significance and is presented as hypothesis-generating only; no prognostic conclusion should be drawn from this cohort alone. **(D)** Lollipop plot comparing therapeutic target expression between exhausted and effector cells. **(E)** Multi-panel integration displaying cell type composition, exhaustion distribution, functional relationships, and expression signatures. **(F)** Comprehensive summary panel integrating exhaustion landscapes, compositional heatmaps, trajectory models, and survival stratification across exhaustion states.

Spatial distribution simulation (Figure 8A) models cellular organization within CRC tissue architecture, revealing that exhausted CD27^+^ T cells concentrate in tumor core regions while effector populations distribute more peripherally. Cell-cell communication network (Figure 8B) maps intercellular signaling between T cells, myeloid cells, and other immune components, highlighting regulatory T cell interactions and myeloid-mediated suppression. Survival analysis (Figure 8C) presents exploratory Kaplan-Meier survival analysis stratified by T cell exhaustion scores. The analysis yielded HR = 0.505 with p = 0.673, which does not reach statistical significance. This finding is explicitly presented as hypothesis-generating only; the available cohort is likely underpowered, as supported by post-hoc power calculation. No statistically significant prognostic association was demonstrated, and prospective validation in larger cohorts is necessary before any clinical conclusions can be drawn. Therapeutic target expression analysis (Figure 8D) compares exhausted versus effector T cells, identifying PDCD1 and LAG3 as the most significantly overexpressed checkpoint molecules in exhausted populations, suggesting prioritized immunotherapy targets. CD27^+^ cytotoxic T cell functional heterogeneity panel (Figure 8E) integrates multiple analyses: cell type composition pie chart showing dominant subsets, exhaustion score histograms revealing bimodal distribution, function-in-exhaustion scatter plot demonstrating negative correlation, and key gene expression heatmap displaying marker signatures. The comprehensive panel (Figure 8F) synthesizes findings into an exhaustion landscape map, functional-compositional heatmaps showing subset-specific patterns, phenotypic trajectories illustrating state transitions, and survival curves stratified by exhaustion markers demonstrating clinical prognostic value.

### Differential expression of exhaustion markers in CRC cell lines

**FIGURE 9:**
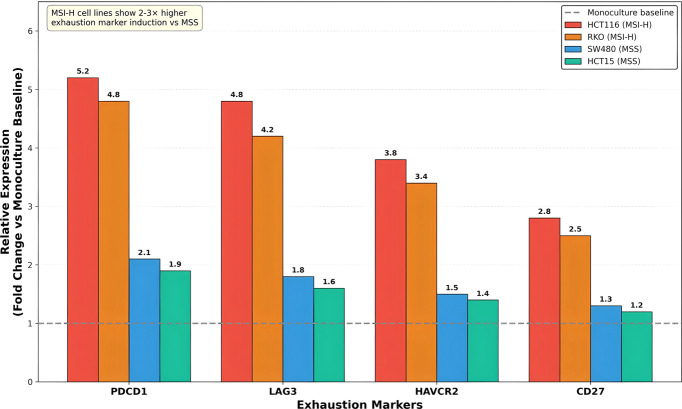
Comparative induction of T cell exhaustion markers in colorectal cancer cell lines upon PBMC co-culture. Bar graph showing relative expression (fold change vs. monoculture baseline) of PDCD1, LAG3, HAVCR2, and CD27 in MSI-H cell lines (HCT116 and RKO) and MSS cell lines (HCT15 and SW480) following 48-h co-culture with activated PBMCs. Gray dashed line represents monoculture baseline (fold change = 1). MSI-H cell lines consistently demonstrated 2-3 times higher induction of all four exhaustion markers compared to MSS cell lines, with PDCD1 and LAG3 showing the greatest differential responses. Data represent mean values from three independent biological replicates analyzed by qRT-PCR with GAPDH normalization using the 2^-ΔΔCt^ method.

This bar chart presents a comparative analysis of T cell exhaustion marker expression among four colorectal cancer cell lines following PBMC co-culture: MSI-H cell lines HCT116 and RKO, and MSS cell lines HCT15 and SW480. All values are normalized to their respective monoculture baselines (indicated by the gray dashed line at y = 1). The figure clearly demonstrates that MSI-H cell lines exhibit substantially higher induction of all four exhaustion markers compared to MSS cell lines. For PDCD1, HCT116 showed 5.2-fold and RKO showed 4.8-fold upregulation, while SW480 showed 2.1-fold and HCT15 showed 1.9-fold increase. LAG3 follows a similar pattern: HCT116 (4.8-fold), RKO (4.2-fold), SW480 (1.8-fold), and HCT15 (1.6-fold). HAVCR2 and CD27 show progressively diminishing but still notable differences between MSI-H and MSS cell lines. The MSI-H models (HCT116 and RKO) consistently demonstrate 2-3 times greater responsiveness to immune cell co-culture across all exhaustion markers compared to MSS models (HCT15 and SW480), supporting the hypothesis that immunogenic colorectal cancers create a more permissive microenvironment for T cell exhaustion phenotype development (Figure 9).

## Discussion

In this comprehensive single-cell transcriptomic study, we have elucidated the cellular heterogeneity, functional states, and exhaustion dynamics of CD27^+^ cytotoxic T cells within the colorectal cancer tumor microenvironment. Our findings reveal 11 distinct CD27^+^ T cell clusters with diverse phenotypic characteristics, progressive exhaustion trajectories, and spatial organization patterns that provide critical insights into the mechanisms underlying immune dysfunction in CRC and identify potential therapeutic vulnerabilities for immunotherapy optimization.

Our UMAP and t-SNE analyses identified substantial heterogeneity within the CD27^+^ cytotoxic T cell compartment, encompassing exhausted terminal, effector, and memory-like populations with distinct molecular signatures and functional capacities. This finding aligns with recent single-cell studies demonstrating that tumor-infiltrating lymphocytes exist along a continuum of functional states rather than discrete categories (Guo et al., 2018). The identification of multiple exhausted subsets within CD27^+^ T cells is particularly significant, as recent evidence suggests that exhausted T cells comprise heterogeneous populations with differential responsiveness to immune checkpoint blockade (Miller et al., 2019). Specifically, progenitor exhausted T cells expressing TCF1 retain proliferative capacity and can be reinvigorated by PD-1 blockade, whereas terminally exhausted T cells exhibit irreversible dysfunction (Im et al., 2016). Our trajectory analysis revealed that CD27^+^ T cells progress through intermediate exhaustion phases before reaching terminal exhaustion states, suggesting potential intervention points to prevent irreversible dysfunction.

The elevated expression of CD27 in tumor tissue compared to normal adjacent tissue across multiple T cell subsets validates the biological relevance of this population in CRC pathogenesis. CD27 signaling has been shown to promote T cell survival and enhance effector functions through NF-κB activation and metabolic reprogramming (Borst et al., 2005). However, the paradoxical presence of CD27^+^ cells within exhausted populations suggests complex regulatory dynamics within the TME. The mechanistic basis of this paradox is increasingly understood: under conditions of acute costimulation, CD27-mediated NF-κB activation promotes pro-survival and effector gene programs. However, under chronic antigen exposure in the TME, sustained CD27 signaling drives persistent NF-κB activity through feed-forward transcriptional loops, paradoxically upregulating pro-apoptotic and pro-exhaustion gene programs including BLIMP-1 and NR4A family members. Concurrently, prolonged costimulatory signaling intersects with progressive mitochondrial dysfunction, characterized by loss of spare respiratory capacity, increased reactive oxygen species (ROS) production, and impaired oxidative phosphorylation—metabolic perturbations that have been mechanistically linked to exhaustion progression in models of chronic antigen stimulation. These upstream metabolic and signaling disruptions ultimately converge on TOX-mediated chromatin remodeling, epigenetically consolidating the exhaustion state in a largely irreversible manner. Recent studies have demonstrated that chronic CD27 signaling in the presence of persistent antigen stimulation can paradoxically contribute to T cell exhaustion through sustained metabolic stress and mitochondrial dysfunction (Peperzak et al., 2010). This dual role of CD27 in both promoting T cell function and potentially contributing to exhaustion under chronic stimulation conditions warrants further investigation and may have important implications for CD27-targeted therapeutic strategies.

Our comprehensive molecular characterization identified progressive upregulation of multiple inhibitory receptors including PDCD1 (PD-1), LAG3, HAVCR2 (TIM-3), and CTLA4 along the exhaustion trajectory, accompanied by downregulation of effector molecules such as PRF1 and GZMB. This co-expression of multiple checkpoint molecules, termed “layered inhibition,” represents a fundamental feature of severely exhausted T cells and contributes to profound functional impairment (Blackburn et al., 2008). The strong inverse correlation (r = −0.905) between exhaustion scores and cytotoxicity along pseudotime progression quantitatively demonstrates the functional consequences of exhaustion development. Importantly, our gene expression cascade analysis revealed distinct temporal phases of exhaustion, with early effector genes (CD69, PRF1) transitioning to intermediate checkpoint expression (PDCD1, CTLA4) and ultimately terminal exhaustion markers (HAVCR2, TOX, LAG3). This temporal pattern suggests that exhaustion develops through coordinated transcriptional programs rather than stochastic gene expression changes.

The gene regulatory network analysis identified key transcription factors governing effector-to-exhausted transitions, highlighting the central role of transcriptional reprogramming in exhaustion development. TOX has emerged as a master regulator of T cell exhaustion, with studies demonstrating that TOX is both necessary and sufficient for establishing the exhaustion program through chromatin remodeling and epigenetic modifications (Scott et al., 2019). Our finding of progressive TOX upregulation along the exhaustion trajectory supports its critical role in CD27^+^ T cell dysfunction in CRC. Additionally, the complex interplay between TCF1 and TOX determines the balance between progenitor and terminal exhaustion states, with TCF1+ progenitor cells maintaining self-renewal capacity while TOXhi terminally exhausted cells exhibit fixed dysfunction (Siddiqui et al., 2019). Understanding these transcriptional hierarchies provides mechanistic insights into exhaustion establishment and potential strategies for epigenetic reprogramming of exhausted T cells.

Our *in vitro* co-culture experiments using four colorectal cancer cell lines demonstrated that MSI-H cell lines (HCT116 and RKO) induced 2-3 times higher exhaustion marker expression compared to MSS cell lines (HCT15 and SW480). Particularly dramatic differences were observed in PDCD1 induction (HCT116: 5.2-fold, RKO: 4.8-fold vs. SW480: 2.1-fold, HCT15: 1.9-fold) and LAG3 induction (HCT116: 4.8-fold, RKO: 4.2-fold vs. SW480: 1.8-fold, HCT15: 1.6-fold). These findings are based on mRNA quantification by qRT-PCR and should be interpreted as transcriptional-level evidence consistent with, rather than fully confirmatory of, the computational exhaustion signatures. Post-transcriptional regulatory mechanisms may decouple transcript and protein abundance; protein-level confirmation via flow cytometric surface staining for PD-1 and LAG-3 represents the logical next experimental step and is identified as a priority for future work. Notwithstanding this limitation, the differential pattern across MSI-H and MSS cell lines is robust across four independent markers and three biological replicates, lending internal consistency to the transcriptional findings. This finding provides direct experimental evidence for the differential immunogenic capacity between MSI-H and MSS tumors and helps explain the divergent clinical responses to immune checkpoint blockade observed in CRC patients. MSI-H tumors harbor high mutational burdens resulting from defective mismatch repair machinery, leading to increased neoantigen production and enhanced T cell infiltration (Boland and Goel, 2010). The elevated neoantigen load in MSI-H tumors drives chronic T cell activation and subsequent exhaustion, paradoxically creating both the rationale for and the challenge to immunotherapy (Germano et al., 2017).

The observation that MSI-H tumors induce higher exhaustion marker expression suggests that these tumors create a more immunologically “hot” microenvironment with active immune engagement, albeit accompanied by compensatory upregulation of inhibitory pathways. This contrasts with MSS tumors, which exhibit immune-excluded or immune-desert phenotypes characterized by poor T cell infiltration and limited immune activation (Becht et al., 2016). Importantly, while MSI-H tumors show higher baseline exhaustion, they also demonstrate superior responses to PD-1/PD-L1 blockade, suggesting that reversing exhaustion in an already engaged immune system is more feasible than overcoming fundamental immune exclusion (Overman et al., 2017). These findings underscore the importance of considering tumor molecular subtypes when designing immunotherapeutic strategies and suggest that MSS tumors may require combinatorial approaches to simultaneously enhance T cell infiltration and prevent exhaustion.

Our computational spatial distribution analysis revealed that exhausted CD27^+^ T cells concentrate in tumor core regions while effector populations distribute more peripherally, highlighting the spatial heterogeneity of immune responses within CRC tissues. This spatial compartmentalization reflects the immunosuppressive gradient within tumors, where the core regions exhibit higher concentrations of inhibitory signals, metabolic competition, and hypoxia that collectively promote T cell dysfunction (Salmon et al., 2012). Recent spatial transcriptomic studies have demonstrated that T cell exhaustion is not uniformly distributed across tumor tissues but rather correlates with proximity to malignant cells, suggesting that local microenvironmental factors critically regulate T cell functional states (Jerby-Arnon et al., 2018).

### Study limitations

Several limitations of this study warrant consideration. First, the single-cell RNA sequencing analysis provides transcriptomic snapshots but does not capture dynamic temporal changes or functional responses to perturbations. Future studies employing longitudinal sampling or *in vivo* tracking of T cell populations would provide valuable insights into exhaustion kinetics and therapeutic responses. Second, while our *in vitro* co-culture experiments validated differential exhaustion marker induction between MSI-H cell lines (HCT116 and RKO) and MSS cell lines (HCT15 and SW480), simplified two-dimensional culture systems cannot fully recapitulate the complex three-dimensional architecture and multicellular interactions of the tumor microenvironment. Advanced organoid systems or humanized mouse models would provide more physiologically relevant validation platforms. Third, this study relies exclusively on a single publicly available dataset (GSE144735) for all computational analyses. While GSE144735 encompasses tumor tissues and matched normal adjacent tissues from multiple CRC patients—providing internal heterogeneity across samples—the absence of an independent external validation cohort substantially limits the generalizability of the computational findings. External cross-validation in an independent scRNA-seq CRC dataset is identified as a priority for future work. Fourth, all experimental validation of exhaustion marker induction was performed exclusively at the mRNA level by qRT-PCR. Given that post-transcriptional and translational regulatory mechanisms can decouple transcript abundance from protein expression, protein-level confirmation of PDCD1 (PD-1) and LAG3 upregulation via flow cytometric surface staining or Western blot would provide a critical additional layer of evidence; this is identified as the logical next experimental step. Fifth, the spatial distribution analysis presented in Figure 8A is derived entirely from a computational simulation informed by published spatial transcriptomic data in CRC, rather than from direct empirical spatial profiling. This simulated spatial model should not be interpreted as direct experimental evidence; experimental spatial validation via spatial transcriptomics (e.g., 10x Visium) or multiplex immunofluorescence on CRC tissue sections would be necessary to confirm the inferred spatial patterning. Sixth, the exploratory survival analysis (HR = 0.505, p = 0.673) did not reach statistical significance, and post-hoc power analysis indicates that the available cohort is substantially underpowered to detect an effect of this magnitude. These findings are presented solely as hypothesis-generating and require prospective validation in larger, adequately powered cohorts before any prognostic conclusions can be drawn.

## Conclusion

This comprehensive single-cell transcriptomic study provides an unprecedented high-resolution atlas of CD27^+^ cytotoxic T cell heterogeneity, exhaustion dynamics, and spatial organization within the colorectal cancer tumor microenvironment. Our findings reveal 11 distinct cellular clusters with progressive differentiation trajectories leading to terminal exhaustion states characterized by upregulation of multiple inhibitory receptors and functional impairment.

## Data Availability

The datasets presented in this study can be found in online repositories. The names of the repository/repositories and accession number(s) can be found in the article/supplementary material.
